# miR-628-3p exacerbates allergic rhinitis inflammation by targeting CELF2: A novel mechanistic insight

**DOI:** 10.1016/j.clinsp.2026.100947

**Published:** 2026-05-01

**Authors:** Min Wang, Yueyan Zhuang, Xiaohui Zhang, Lijuan Li

**Affiliations:** a Health Management Center, The 964th Hospital of PLA Joint Logistic Support Force, Changchun City 130062, Jilin Province, China; b Departments of Otolaryngology, The 964th Hospital of PLA Joint Logistic Support Force, Changchun City 130062, Jilin Province, China; c Departments of Outpatient, The 964th Hospital of PLA Joint Logistic Support Force, Changchun City 130062, Jilin Province, China

**Keywords:** miR-628-3p, CELF2, Allergic rhinitis, Nasal epithelial cells, Inflammation

## Abstract

**Background:**

Allergic Rhinitis (AR) represents a widespread chronic inflammatory condition, with especially prevalence observed in children and urban populations.

**Aim:**

This study primarily explored the expression of miR-628–3p in AR and its functional implications on the inflammatory response, and elucidated the underlying molecular mechanisms through which miR-628–3p regulates AR.

**Methods:**

RT-qPCR analysis quantified miR-628–3p expression in AR patient serum and cell models. The biological functionality of the cells was assessed using the CCK-8 assay and flow cytometry. Inflammatory cytokine levels were measured via ELISA. Bioinformatic prediction and dual-luciferase reporter assays were employed to validate miR-628–3p targets.

**Results:**

miR-628–3p expression was upregulated in the serum and cell samples of AR compared to healthy controls. miR-628–3p can target and bind to the downstream CELF2. Downregulation of miR-628–3p reduced apoptosis in AR cells and suppressed levels of inflammatory factors, while transfection of si-CELF2 reversed the protective ability of miR-628–3p inhibitor on AR cells.

**Conclusions:**

Knockdown of miR-628–3p may contribute to the pathogenesis of AR by mediating CELF2 to reduce the apoptosis and inflammatory response of AR cells, suggesting its potential protective role against AR-related cellular damage.

## Highlights

• First evidence that miR-628–3p is upregulated in AR patients and cell models.
• miR-628–3p promotes inflammatory response and apoptosis in nasal epithelial cells.
• Mechanistic validation shows CELF2 is a direct target of miR-628–3p.
• Knockdown of miR-628–3p alleviates inflammation via CELF2 upregulation.
• Identifies miR-628–3p/CELF2 axis as a potential therapeutic target for AR.

## Introduction

Allergic Rhinitis (AR) is clinically characterized as a chronic, noninfectious inflammatory disorder predominantly mediated by Immunoglobulin E (IgE), which arises following allergen exposure to the nasal mucosa, with symptoms such as nasal congestion, pruritus, and paroxysmal sneezing.^1^^,^^2^ The escalating prevalence of AR correlates significantly with progressive urbanization and deteriorating ambient air quality.^3^ Global epidemiological data indicate that approximately 30% of adults are affected by AR, with children exhibiting considerably higher incidence rates.^4^ Prolonged and recurrent episodes of AR may lead to complications such as asthma and chronic sinusitis, which have detrimental effects on the daily activities and economic productivity of patients.^5^ Therefore, elucidating the molecular mechanisms of AR and identifying novel biomarkers represent priorities for advancing therapeutic strategies.

MicroRNAs (miRNAs) are a family of non-coding RNAs that bind to target gene mRNAs to form miRNA-induced silencing complexes that initiate or inhibit translation.^6^ In recent years, there has been a gradual increase in reports of miRNAs, underscoring their pivotal role in the regulation of cellular functions, such as stress and immune responses.^7^ It has been demonstrated that miRNAs are implicated in the pathogenesis of many allergic diseases, including AR.^8^ Overexpression of miR-126 affects the occurrence and progression of AR by regulating the function of Th cells and the expression of pertinent cytokines.^9^ miR-150–5p was upregulated in AR patients and obviously elevated the levels of Th2 cells and ILC2 differentiation, which may serve as a potential target for AR therapeutics.^10^ miR-628–3p demonstrates dysregulated expression across multiple pathological conditions, including cancer, autoimmune disorders, and inflammatory diseases.^11^^,^^12^ In patients with fractures, miR-628–3p was observed to be upregulated.^13^ Interestingly, miR-628–3p was revealed to be positively expressed in atopic dermatitis.^14^ However, whether miR-628–3p is involved in regulating the pathogenesis of AR is unknown and remains to be further investigated.

Human Nasal Epithelial Cells (HNEPCs) are the physical barrier of the nasal cavity and participate in the pathophysiologic process of AR by mediating mechanisms such as immunity and neuromodulation.^15^ In light of these functions, HNEPCs were selected in this study, and AR cell models were established through IL-13 induction to facilitate in vitro cellular assays.

The authors postulate that dysregulation of miR-628–3p contributes to AR pathogenesis and inflammatory modulation. To substantiate this hypothesis, the present study quantified miR-628–3p levels in serum and cellular models of AR patients and verified the modulation of HNEPCs activity and inflammatory responses by dysregulated miR-628–3p The downstream targets of miR-628–3p were further predicted, and their impact on AR progression was revealed. An in-depth understanding of the role of miR-628–3p in AR is anticipated to provide valuable insights and a scientific basis for early intervention in patients to control the inflammatory response.

## Materials and methods

### AR patients and serum sample collection

This study was performed in accordance with the principles of the Declaration of Helsinki. With the permission of the Ethics Committee of The 964th Hospital of PLA Joint Logistic Support Force, (2023Y0010). Informed consent was obtained from all individual participants included in the study. The study follows the STARD guidelines. The authors recruited 120 AR patients admitted to our hospital (AR group) and 120 healthy volunteers (healthy control group). Patients were included if they presented with two or more of the following typical symptoms of AR for at least one hour on most days: nasal itching, sneezing, rhinorrhea, and nasal congestion. The clinical diagnosis was confirmed by specialist physicians based on these symptoms, along with positive skin prick tests or elevated serum specific IgE levels to common aeroallergens. Patient data were complete, and no pharmacological interventions were performed prior to admission. Patients with asthma, atopic diseases, autoimmune diseases, and malignant tumors were also excluded.

Blood samples were collected from both study groups and serum samples were obtained by routine centrifugation. A portion of these samples was analyzed for clinical indicators (recorded in Table 1), and the other was stored in a −80 °C refrigerator for backup.

**Table 1:** Comparison of general data of the included subjects.

Indicators	HC group (n = 120)	AR group (n = 120)	p-value
Gender, n (%)			0.700
Male	60 (50.00)	63 (52.50)	
Female	60 (50.00)	57 (47.50)	
Age (years)	21.29±6.92	20.69±4.96	0.447
IgE (IU/mL)	20.53±5.85	201.63±31.88	<0.001
NEU (10^9^/L)	3.54±1.01	3.60±0.77	0.570
WBC (10^9^/L)	6.63±1.32	7.60±1.43	<0.001
LYM (10^9^/L)	2.09±0.56	2.51±0.80	<0.001
EO (10^9^/L)	0.12±0.04	0.41±0.15	<0.001
BASO (10^9^/L)	0.03±0.02	0.04±0.03	<0.001
IL-4 (pg/mL)	24.90±2.45	124.15±6.36	<0.001
IL-5 (pg/mL)	49.66±6.61	238.67±23.05	<0.001
IL-13 (pg/mL)	25.53±4.96	138.52±10.48	<0.001

HC, Healthy Control; AR, Allergic Rhinitis; IgE, Immunoglobulin E; NEU, Neutrophils; WBC, White Blood Cell; LYM, Lymphocytes; EO, Eosinophils; BASO, Basophils; IL-4, Interleukin-4; IL-5, Interleukin-5; IL-13, Interleukin-13.

### Acquisition and cultivation of cells

HNEPCs were purchased from the Cell Bank of the Chinese Academy of Sciences (Shanghai, China). HNEPCs were inoculated in RPMI-1640+ fetal bovine serum (10%; FBS, Gibco, USA) medium and placed in a humidified incubator at 37 °C for culture.

To obtain AR cell models in vitro, HNEPCs were induced with recombinant human IL-13 (SRP3274; 50 ng/mL, purity > 98%; Sigma-Aldrich, St. Louis, MO, USA) and maintained for 24 h.^16^

### Transfection of cells

The incubated HNEPCs were transferred to 6-well plates at a density of 5 × 10^5^ cells/well, and allowed to reach 60%‒70% confluence at the time of transfection. Lipofectamine 2000 reagent (Invitrogen, Carlsbad, CA, USA) was added to each well. Transfected cells were obtained after 48 h of treatment, and transfection efficiency was verified by RT-qPCR assays. GenePharma (Shanghai, China) synthesized miR-628–3p inhibitor, miR-628–3p mimic, and si-CELF2 required for transfection.

### Proliferation and apoptosis of cells

Cells were inoculated in 96-well plates, and three parallel wells were set up for each group (technical replicates) and then incubated overnight. CCK-8 solution (10 μL; KeyGEN, Nanjing, China) was supplemented in each well and incubated under dark conditions for 2 h. The absorbance was finally evaluated by a microplate reader (450 nm; Eppendorf, Germany) after 48 h of further maintenance.

Cells were washed with PBS solution and then resuspended in RPMI-1640 medium. FITC-Annexin V and PI (5 μL each; BD Biosciences, San Jose, CA, USA) were transferred to the cells and treated in the dark for 15 min. The necrotic cells were distinguished by a flow cytometry (BD Biosciences, San Jose, CA, USA).

All experiments were independently performed at least three times (biological replicates, n = 3).

### ELISA for the concentration of inflammatory factors

The levels of inflammatory factors in the cells were confirmed by Human IL-4/IL-5/IL-13 ELISA Kit (Solarbio, China), respectively.

### RT-qPCR

RNA was taken from serum and HNEPCs samples using Trizol reagent (Invitrogen, Carlsbad, CA, USA). Assessment of RNA concentration and purity was conducted via a NanoDrop 2000 spectrophotometer (Thermo Fisher Scientific, Waltham, MA, USA). cDNA was then obtained by reverse transcription assay (SuperScript IV Reverse Transcriptase kit; Thermo, USA). The cDNA was mixed with the reaction reagents in the SYBR Green PCR kit (Qiagen, Hilden, Germany) and amplified by PCR, with U6 as the reference gene for miR-628–3p The derived data were subjected to 2^-ΔΔCt^ to calculate the results of the final reaction.

### Western blot

Total protein was extracted and quantified from HNEPCs by the BCA kit (Sigma, USA). The protein was separated by SDS-PAGE and transferred to PVDF membrane. After blocking, membranes were incubated with primary antibodies at 4 °C overnight and with secondary antibodies for 2 h at room temperature. The band grayscale was analyzed by the ImageJ software.

### Prediction of miR-628–3p target genes

The downstream targets of miR-628–3p were screened by TargetScan and miRDB databases, and Venn diagram was made to reflect the prediction results. After further comparison of possible targets, the binding sites between miR-628–3p and CELF2 were analyzed through TargetScan database.

### Luciferase reporter gene assay

Wild-type or mutant CELF2 plasmids (WT-CELF2/MUT-CELF2) were constructed by GenePharma (Shanghai, China), and miR-628–3p inhibitor or mimic was co-transfected into HNEPCs using Lipofectamine 2000 reagent. After 48 h of treatment, the luciferase activity of the cells was quantified through the Dual-Luciferase Reporter Assay Kit (Promega, Madison, WI, USA).

### Statistical analysis

Data were processed and plotted using GraphPad Prism software (version 9.0; CA, USA). The required sample size for the experiment was determined using G*Power software (version 3.1; Germany). The measurement data were expressed as mean ± standard deviation, the normality of the distribution was assessed using the Shapiro-Wilk test, and the homogeneity of variances was confirmed using Levene’s test. For comparisons involving more than two groups, the authors used one-way analysis of variance (ANOVA) followed by Tukey's post hoc test. For comparisons involving only two groups, the independent samples *t*-test was used. The count data were expressed as the n of cases (%), and differences between groups were verified by chi-square analysis. Statistical significance was indicated at p < 0.05.

## Results

### Analysis of clinical data

In Table 1, there were 60 cases of both males and females, aged 21.29 ± 6.92 years in the recruited HC group. In the AR group of subjects, 63 males and 57 females were recruited with an age of 20.69 ± 4.96 years. The gender and age distributions of the two cohorts were analyzed, revealing no statistically significant differences (p > 0.05). In addition, biochemical markers (including IgE, WBC, LYM, EO, BASO) and inflammatory factors (such as IL-4, IL-5, IL-13) levels were upregulated in AR patients compared to HC group (p < 0.001). This is suggestive of the inflammation that often accompanies AR patients.

### Changes in miR-628–3p levels in AR

**Fig. 1:**
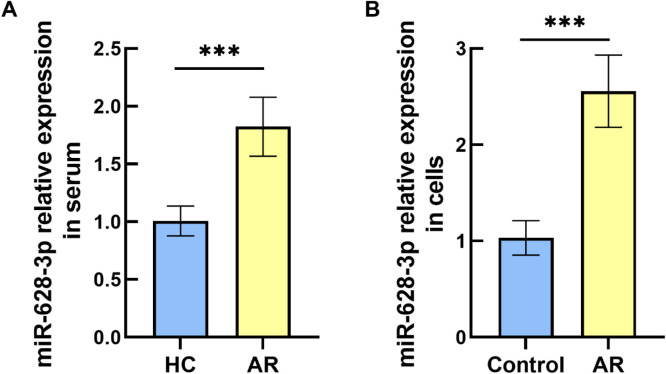
Quantitative analysis of miR-628–3p expression. (A) Serum miR-628–3p was enriched in AR patients compared to healthy controls. (B) miR-628–3p level was upregulated in AR cell models compared to control cells. Data are presented as the mean ± SD from independent biological experiments. Statistical significance was determined by *t*-test. ***p < 0.001 vs. control.

Serum miR-628–3p levels in AR were markedly enhanced compared to HC, as verified by RT-qPCR analysis (Fig. 1A, p < 0.001). Similarly, miR-628–3p expression was also elevated in AR cell models compared to control HNEPCs (Fig. 1B, p < 0.001). This states that overexpression of miR-628–3p is associated with the appearance of AR.

### Inflammatory responses are suppressed by miR-628–3p inhibitor

**Fig. 2:**
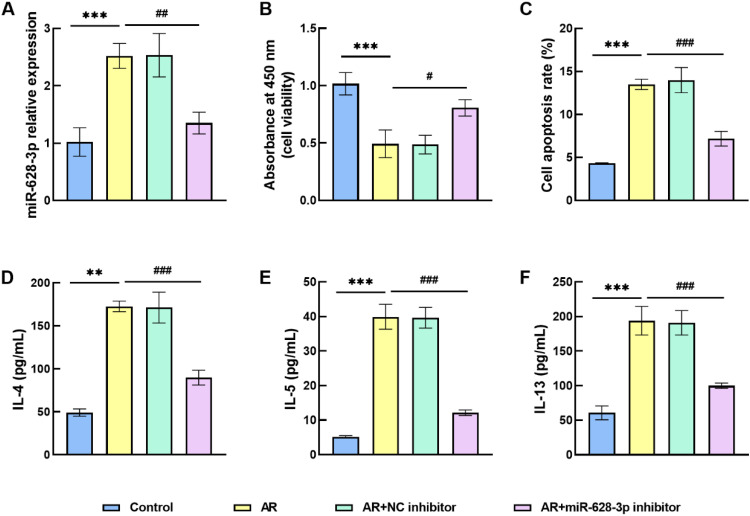
Effect of miR-628–3p on cell function and inflammation levels. (A) Transfection efficiency of the miR-628–3p inhibitor was confirmed by RT-qPCR assay. (B) AR cell proliferation was elevated after miR-628–3p levels were suppressed. (C) The apoptosis rate of AR cells decreased with the downregulation of miR-628–3p levels. (D‒F) The concentrations of inflammatory factors were decreased after miR-628–3p inhibitor intervention. Data are presented as the mean ± SD from independent biological experiments. Statistical significance was determined by one-way ANOVA followed by Tukey’s post-hoc test for multiple comparisons. ** p < 0.01, *** p < 0.001 vs control; ^#^ p < 0.05, ^##^ p < 0.01, ^###^ p < 0.001 vs. AR.

Transfection experiments were performed in HNEPCs, and Fig. 2A reflects the upregulation of miR-628–3p levels in AR cells, which were significantly suppressed after silencing miR-628–3p by transfection. Assays, including the CCK-8 proliferation assay and apoptosis analysis, indicated a marked decrease in cell proliferation within the AR cell model, accompanied by an increase in the apoptosis rate. Conversely, the biological function of the cells was restored following the knockdown of miR-628–3p (Fig. 2B‒C). The above results suggest that silencing miR-628–3p enhances the viability of AR cells. Moreover, assessment of inflammatory factor concentrations revealed that levels of IL-4, IL-5, and IL-13 were significantly elevated in the induction-acquired AR cell model, while IL-4, IL-5, and IL-13 levels were also downregulated when miR-628–3p was inhibited (Fig. 2D‒F). These results imply that the miR-628–3p inhibitor alleviated the inflammatory response in AR cells.

### CELF2 is a downstream target gene of miR-628–3p

**Fig. 3:**
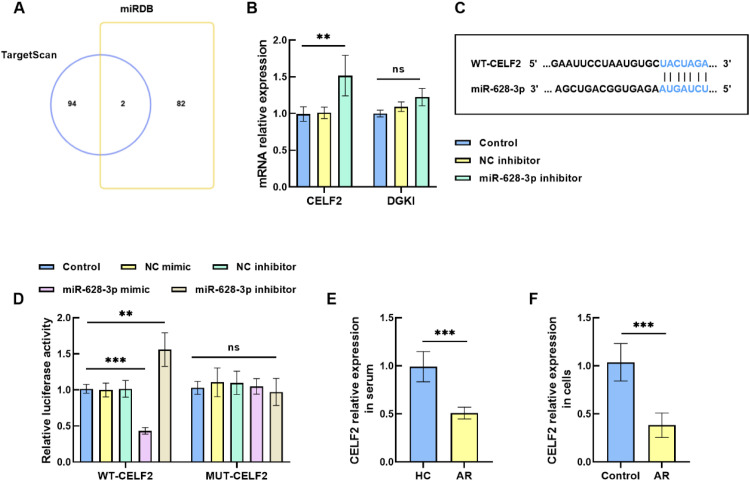
Interaction between miR-628–3p and CELF2. (A) Venn diagram of miR-628–3p target gene. (B) CELF2 and DGKI levels after miR-628–3p knockdown. (C) There were complementary sequences between miR-628–3p and CELF2. (D) Luciferase activity assay to validate the relationship between miR-628–3p and CELF2. (E‒F) CELF2 level was diminished in the AR. Data are presented as the mean ± SD from independent biological experiments. Statistical significance was determined by *t*-test or one-way ANOVA followed by Tukey’s post-hoc test. ^ns^ p > 0.05, ** p < 0.01, *** p < 0.001 vs. control.

The online databases TargetScan and miRDB jointly predicted two downstream targets of miR-628–3p and generated a Venn diagram to illustrate their findings (Fig. 3A). Functional validation via transfection assays revealed that silencing miR-628–3p upregulated CELF2 level, while exerting no significant effect on DGKI level (Fig. 3B). Furthermore, TargetScan prediction confirmed that complementary binding sites of CELF2 and miR-628–3p may have targeting relationships (Fig. 3C). The luciferase activity of the WT-CELF2 group was downregulated following transfection with miR-628–3p mimic and upregulated after transfection with miR-628–3p inhibitor. However, the modulation of luciferase activity by miR-628–3p mimic/inhibitor in the MUT-CELF2 group was not statistically significant (Fig. 3D). These results imply that CELF2 serves as a downstream direct target of miR-628–3p Additionally, serum levels of CELF2 were found to be diminished in AR patients compared to the HC group (Fig. 3E). Decreased CELF2 levels were also considered in the constructed AR cell model compared to control HNEPCs (Fig. 3F).

### Inflammatory response mediated by silencing CELF2

**Fig. 4:**
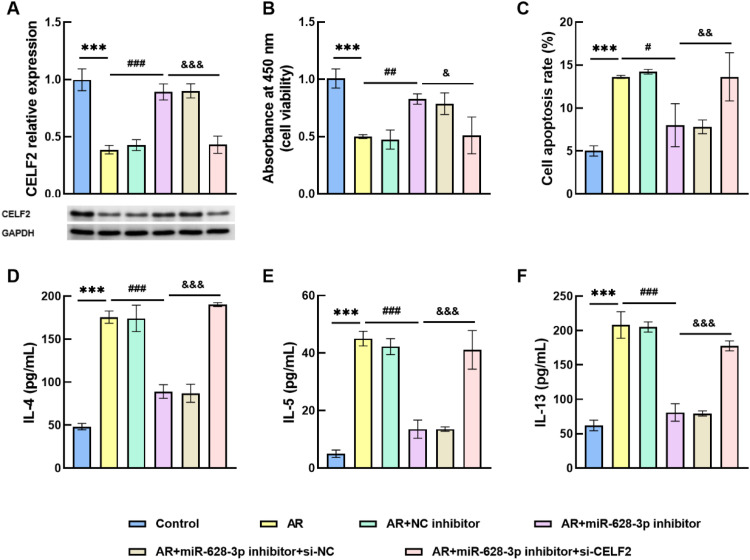
Silencing of CELF2 regulates cell viability and inflammatory responses. (A) Transfection efficiency of miR-628–3p inhibitor and si-CELF2 in AR cells by RT-qPCR and Western blot assay. (B‒C) Changes in cell proliferation and apoptosis. (D‒F) si-CELF2 counteracted the inhibitory effect of miR-628–3p knockdown on inflammatory factor secretion in AR cells. Data are presented as the mean ± SD from independent biological experiments. Statistical significance was determined by one-way ANOVA followed by Tukey’s post-hoc test for multiple comparisons. *** p < 0.001 vs. control; ^#^ p < 0.05, ^##^ p < 0.01, ^###^ p < 0.001 vs. AR; ^&&^ p < 0.01, ^&&&^ p < 0.001 vs. AR+miR-628–3p inhibitor.

For in vitro cell experiments, the authors co-transfected si-CELF2 and miR-628–3p inhibitor into HNEPCs. In the AR cell model, CELF2 exhibited a marked basal suppression, with miR-628–3p knockdown inducing its significant upregulation, and CELF2 silencing effectively normalized expression levels (Fig. 4A). Notably, the knockdown of miR-628–3p conferred a protective effect on AR cells, although this beneficial outcome was counteracted by the si-CELF2 intervention (Fig. 4B). Meanwhile, miR-628–3p inhibitor reduced apoptosis in transfected cells, whereas si-CELF2 reversed the ability of dysregulated miR-628–3p to inhibit apoptosis in AR cells (Fig. 4C). Moreover, low levels of miR-628–3p suppressed the reduced levels of inflammatory cytokines such as IL-4 (Fig. 4D), IL-5 (Fig. 4E), and IL-13 (Fig. 4F), while co-transfection of si-CELF2 reversed the impact of miR-628–3p on the inflammatory response. The above experimental findings inferred that miR-628–3p repaired the proliferative activity of AR cell models and controlled the exacerbation of inflammatory responses by targeting CELF2.

## Discussion

The pathogenesis of AR is the type I allergic reaction mediated by allergen-specific IgE, which is a prevalent chronic immune-inflammatory disease.^17^ Current clinical treatments employ a stepwise and personalized approach, with nasal corticosteroids and antihistamines forming the cornerstone of therapy, and immunotherapy and surgical interventions may also be new options.^18^ Nevertheless, AR is characterized by intermittent and persistent morbidity.^19^ Patients not only require year-round medication for recurrent symptoms but also may develop complications such as asthma. Consequently, the exploration of noninvasive biomarker therapies holds promise as an innovative strategy for managing AR.

Previous evidence supports the importance of miRNA regulation in AR, but its molecular mechanisms remain incompletely elucidated. Fatma et al. have proposed that miRNAs are differentially expressed in patients with varying forms of AR, and that miR-181a, miR-125b and miR-206 have diagnostic value in predicting disease risk.^20^ miR-135a is also described in the recent literature as dysregulated in AR patients and shows potential as a diagnostic tool.^21^ Through quantitative analysis of samples from AR patients and the healthy control population in this study, the authors realized that serum miR-628–3p was upregulated in AR. Similarly, miR-628–3p exhibited enriched expression in the AR cell models constructed using HNEPCs. The high level of miR-628–3p in the plasma of patients with atopic keratoconjunctivitis, as well as its prominent expression in Stevens-Johnson syndrome were confirmed by Mayumi et al.,^14^^,^^22^ and these reports support these findings.

The inflammatory response plays a central role in the development and progression of AR, with a predominance of Th2-type responses, including IL-4, IL-5, and IL-13.^23^ Among these, IL-4 promotes and activates IgE production and induces Th2 cell differentiation; IL-5 mediates the differentiation and survival of eosinophils and aggravates nasal mucosal inflammation; and IL-13 stimulates mucus secretion and disrupts the epithelial barrier.^24^^,^^25^ The authors observed that in the in vitro cellular assays, the onset of AR inhibited the activity of HNEPCs, resulting in increased apoptosis and a substantial release of inflammatory mediators. However, when transfected with miR-628–3p inhibitor, the apoptosis rate and inflammatory markers of AR cells were prominently downregulated, indicating that the downregulation of miR-628–3p may alleviate AR by attenuating the inflammatory response.

CELF proteins are thought to be associated with neurologic, cardiac and skeletal muscle-like diseases. As a core member of this family, CELF2 is located on chromosome 10p13–14 and has been mostly shown to be downregulated in various tumors.^26^, ^27^, ^28^ Michael and colleagues demonstrated an interaction between CELF2 and hnRNP C that modulates T cell activity.^29^ In this study, CELF2 expression was diminished in the AR, and it was validated as a direct downstream target of miR-628–3p by online databases and luciferase assays. In Liao's study, miR-20a stimulated glioma cell proliferation while concurrently inhibiting apoptosis, while silencing CELF2 reversed the effect of miR-20a level.^30^ The role of silencing CELF2 was similarly reflected in the present study, where the authors noted that the miR-628–3p inhibitor stimulated the biological activity of AR cells, whereas si-CELF2 suppressed this function. Meanwhile, transfection of si-CELF2 led to an upregulation of the content of inflammatory mediators in AR cells, which reflected that knockdown of miR-628–3p and CELF2 induced apoptosis of HNEPCs and intensified the inflammatory response associated with AR.

The limitations existed in this study: First, the clinical sample size of AR patients is insufficient, and more patient samples need to be collected for corroboration. Second, IL-13-induced cell models cannot fully mimic the pathological environment of AR and animal models need to be constructed according to the experimental purpose for in vivo verification. Third, clinical correlation studies or broader screening of miR-628–3p in AR populations.

## Conclusions

The present findings provide the first evidence that miR-628–3p is upregulated in AR patients and cellular models. miR-628–3p promotes inflammatory responses and apoptosis in nasal epithelial cells. Mechanistic validation shows CELF2 is a direct target of miR-628–3p In summary, knockdown of miR-628–3p alleviates inflammation by upregulating CELF2, identifying the miR-628–3p/CELF2 axis as a potential therapeutic target for AR.

## Data availability statement

To protect patient privacy and comply with medical data confidentiality guidelines, all raw patient-related data have been securely stored. All basic study data are available from the corresponding author upon reasonable request.

## Authors’ contributions

All authors contributed to the study conception and design. Study concept and design: M. W., Y.Y. Z., and L.J. L.; analysis and interpretation of data: M. W., Y.Y. Z., and X.H. Z.; drafting of the manuscript: M. W., and Y.Y. Z.; critical revision of the manuscript for important intellectual content: L.J. L.; statistical analysis: M. W., Y.Y. Z., and X.H. Z.

## Funding

The authors declare that no funds, grants, or other support were received during the preparation of this manuscript.

## Declaration of competing interest

The authors report there are no competing interests to declare.
